# Data on records of environmental phenomena using low-cost sensors in vineyard smallholdings

**DOI:** 10.1016/j.dib.2020.106524

**Published:** 2020-11-18

**Authors:** Sergio Trilles, Alberto González-Pérez, Benito Zaragozí, Joaquín Huerta

**Affiliations:** 1 Institute of New Imaging Technologies, Universitat Jaume I, Av. Vicente Sos Baynat s/n, Castelló de la Plana, Spain; 2 Departament de Geografia, Universitat Rovira i Virgili, C/Joanot Martorell, Vilaseca, Spain

**Keywords:** Internet of Things, Open hardware, Low-cost sensors, Smart farming, Vineyard smallholdings

## Abstract

Inadequate weather conditions are one of the main threats to the correct development of sensitive crops, where a bad situation can lead to greater stress on plants and their weakness against various diseases. This statement is especially decisive in the cultivation of the vineyard. Meteorological monitoring of vineyard parcels is vital to detect and prevent possible fungal diseases. The development of new Information and Communication Technologies, linked to the Smart Farming movement, together with the reduced cost of electronic components, have favoured a greater availability of meteorological monitoring stations to get to know first-class hand the state of the vineyard smallholdings. This work provides a set of over 750,000 environmental raw data records collected by low-cost Internet of Things nodes, primarily located within vineyard smallholdings. The published observations were collected between 2018-04-01 and 2018-10-31 and were validated in previous research to determine the data's reliability.

## Specifications Table

**Table:** 

Subject	Computer Networks and Communications, Engineering
Specific subject area	Application of computing networks and engineering in monitoring environmental phenomena in vineyard smallholdings using IoT nodes.
Type of data	Text files (Comma Separated Values)
How data were acquired	Data were captured using low-cost sensors from *SEnviro* nodes
Data format	Raw sensor data
Parameters for data collection	The IoT nodes were deployed within vineyard smallholdings and in outdoor environments for testing
Description of data collection	The data set was collected from 2018-04-01 to 2018-10-31. This data set contains environmental sensor measurements, such as temperature, air humidity, soil moisture, atmospheric pressure, rain, and wind speed/direction sensors. Battery level values are also included.
Data source location	Each IoT node has a different place. All locations are provided in the manuscript.
Data accessibility	Dataset: https://doi.org/10.5281/zenodo.3727310 Source code: http://doi.org/10.5281/zenodo.4229257
Related research article	Trilles, S.; González-Pérez, A.; Huerta, J. An IoT proposal for monitoring vineyards called *SEnviro* for agriculture. In Proceedings of the 8th International Conference on the Internet of Things (IoT ’18). Association for Computing Machinery, New York, NY, USA, Article 20, 1–4. https://doi.org/10.1145/3277593.3277625

## Value of the Data

• The data set presented in this paper can be used for further experiments to detect diseases influenced by meteorological conditions, such as black rot, downy and powdery mildews and botrytis [1].
• It can also be used to validate the low-cost sensors included in this development using professional weather stations. Also, the low-cost sensors can be compared to other low-cost sensors, and a qualitative and performance comparative can be carried out. Data has been kept raw (including outliers), thus allowing sensor reliability to be verified.
• Another value of this data set is in the development of energy strategies. The battery levels of IoT nodes are also provided; these values can be used to study how meteorological variables affect the discharge of the battery and its charge using a solar panel.

## 1 Data Description

The data set is published online in the Zenodo data repository [2]. The presented data were collected between 2018-04-01 and 2018-10-31 using a set of low-cost IoT nodes called *SEnviro* [3]. *SEnviro* nodes feature different improvements, such as the feasibility of performing remote updates managing Over-the-Air (OTA) updates; a greater autonomy supporting 3G connectivity, and solar panel plus applied energy policies; and replicability because it is made up of open hardware and other elements such as 3D-printed pieces [4]. Each *SEnviro* node collects environmental sensor measurements, such as temperature, air humidity, soil moisture, atmospheric pressure, rain, and wind speed/direction phenomena. Battery level values are also recorded. Seven *SEnviro* nodes were deployed in outdoor environments; four of them were located within vineyard smallholdings. The following tables (Tables 1–8) examine summary statistics for each phenomenon and report the number of measurements, mean, standard deviation, median, minimum and maximum. Table 7 shows the summary of the wind direction phenomenon since this observation is qualitative, the mode value is shown instead of the median, maximum and minimum values. In order to distinguish which node has generated the observations, an identifier has been used.

**Table 1:** Summary of the atmospheric pressure phenomenon.

Atmospheric pressure phenomenon			
*SEnviro* identifier	Count	Mean (STD) (hPa)	Median [min, max] (hPa)
270043001951343334363036	19358	990.27 (2.86)	990.32 [983.58, 999.54]
380033001951343334363036	15690	978.22 (2.11)	978.19 [974.43, 982.71]
46005a000351353337353037	4332	988.47 (2.15)	988.40 [984.46, 992.97]
4e0022000251353337353037	1815	1008.83 (2.94)	1009.43 [1005.13, 1013.54]
4e0031000251353337353037	16227	988.71 (2.08)	988.77 [984.85, 993.17]
46004e000251353337353037	4522	983.71 (1.70)	983.77 [980.21, 986.95]
200034001951343334363036	18654	1004.51 (2.44)	1004.67 [999.11, 1009.03]

**Table 2:** Summary of the temperature phenomenon.

Temperature phenomenon			
*SEnviro* identifier	Count	Mean (STD) (°C)	Median [min, max] (°C)
270043001951343334363036	15036	23.24 (5.95)	22.57 [6.86, 34.46]
380033001951343334363036	15917	26.32 (5.39)	25.56 [17.90, 36.52]
46005a000351353337353037	4470	25.10 (5.39)	23.38 [17.51, 36.15]
4e0022000251353337353037	228	14.74 (5.60)	16.85 [3.21, 20.29]
4e0031000251353337353037	16562	25.83 (6.19)	24.52 [16.21, 37.53]
46004e000251353337353037	4591	23.56 (5.95)	22.99 [14.03, 34.33]
200034001951343334363036	18564	26.62 (5.90)	26.09 [15.73, 38.09]

**Table 3:** Summary of the humidity phenomenon.

Humidity phenomenon			
*SEnviro* identifier	Count	Mean (STD) (RH)	Median [min, max] (RH)
270043001951343334363036	15832	60.27 (15.24)	60.35 [34.16, 96.20]
380033001951343334363036	15882	54.05 (14.92)	53.10 [30.44, 80.14]
46005a000351353337353037	4555	74.16 (12.30)	76.16 [48.08, 92.51]
4e0022000251353337353037	1919	67.72 (20.18)	70.53 [66.92, 96.90]
4e0031000251353337353037	16284	56.73 (14.63)	56.85 [31.15, 81.79]
46004e000251353337353037	4581	52.21 (13.94)	52.26 [26.76, 78.65]
200034001951343334363036	18693	56.36 (15.10)	55.47 [30.29, 82.95]

**Table 4:** Summary of the precipitation phenomenon.

Precipitation phenomenon			
*SEnviro* identifier	Count	Mean (STD) (mm)	Median [min, max] (mm)
270043001951343334363036	20145	0.001 (0.02)	0.00 [0.00, 1.39]
380033001951343334363036	19810	0.01 (0.20)	0.00 [0.00, 14.81]
46005a000351353337353037	5820	0.006 (0.082)	0.00 [0.00, 3.07]
4e0022000251353337353037	2819	0.02 (0.34)	0.00 [0.00, 15.37]
4e0031000251353337353037	21707	0.01 (0.19)	0.00 [0.00, 16.20]
46004e000251353337353037	5713	0.01 (0.17)	0.00 [0.00, 7.26]
200034001951343334363036	22703	0.01 (0.15)	0.00 [0.00, 3.58]

**Table 5:** Summary of the soil moisture phenomenon.

Moisture phenomenon			
*SEnviro* identifier	Count	Mean (STD) (%)	Median [min, max] (%)
270043001951343334363036	15311	38.00 (8.00)	38.00 [20.00, 55.00]
380033001951343334363036	15522	67.00 (17.00)	69.00 [34.00, 87.00]
46005a000351353337353037	4318	58.00 (21.00)	63.00 [9.00, 82.00]
4e0022000251353337353037	805	65.00 (7.00)	65.00 [50.00, 75.00]
4e0031000251353337353037	16258	89.00 (0.00)	89.00 [87.00, 92.00]
46004e000251353337353037	4428	22.00 (31.00)	0.00 [0.00, 79.00]
200034001951343334363036	17565	20.00 (4.00)	17.00 [16.00, 31.00]

**Table 6:** Summary of the wind speed phenomenon.

Wind speed phenomenon			
*SEnviro* identifier	Count	Mean (STD) (km/h)	Median [min, max] (km/h)
270043001951343334363036	8986	4.29 (1.92)	4.82 [2.19, 7.24]
380033001951343334363036	12566	4.81 (2.55)	4.82 [2.19, 12.04]
46005a000351353337353037	2100	3.22 (1.14)	2.41 [2.41, 7.23]
4e0022000251353337353037	1458	4.54 (2.57)	3.50 [1.75, 10.51]
4e0031000251353337353037	6695	3.34 (1.18)	2.41 [2.40, 7.23]
46004e000251353337353037	1766	3.36 (1.20)	2.41 [2.40, 7.23]
200034001951343334363036	15889	3.33 (1.58)	2.08 [1.08, 7.60]

**Table 7:** Summary of the wind direction phenomenon.

Wind direction phenomenon			
*SEnviro* identifier	Count	Mean (STD) (0-7)	MODE (0-7)
270043001951343334363036	19919	3.84 (2.21)	5.0
380033001951343334363036	19756	2.94 (2.54)	3.0
46005a000351353337353037	5817	2.82 (2.74)	0.0
4e0022000251353337353037	2475	3.56 (2.97)	7.0
4e0031000251353337353037	21671	2.26 (2.09)	3.0
46004e000251353337353037	5697	2.60 (2.47)	-1.0
200034001951343334363036	15388	2.60 (2.58)	1.0

**Table 8:** Summary of the IoT node battery level.

IoT node battery level			
*SEnviro* identifier	Count	Mean (STD) (%)	Median [min, max] (%)
270043001951343334363036	19643	66.04 (16.43)	70.12 [0.00, 100.19]
380033001951343334363036	19368	69.34 (16.03)	71.76 [0.00, 98.93]
46005a000351353337353037	5793	60.83 (21.85)	66.58 [0.45, 87.21]
4e0022000251353337353037	2785	53.01 (22.67)	57.26 [0.00, 96.55]
4e0031000251353337353037	21423	68.04 (12.99)	68.97 [2.41, 91.65]
46004e000251353337353037	5553	65.05 (17.18)	66.43 [0.00, 99.56]
200034001951343334363036	21996	68.68 (13.91)	71.02 [6.61, 97.66]

Each *SEnviro* node transmitted all the measurements every 10 minutes to a main server in real-time. The resulting data set has been published as raw data, so this means that invalid or missing values may appear. These could have been caused by the low-quality sensors themselves, the lack of 3G coverage or other node deployment failures [5]. Finally, it should be mentioned that not all IoT nodes have been deployed throughout the aforementioned period.

Two studies were carried out to determine the reliability of the data obtained by different *SEnviro* nodes installed in vineyards [6]. The temporal behaviour of data obtained in the *SEnviro* stations was compared with professional and official stations in the same area in order to study their homogeneity and to identify possible difficulties and differences for possible implementation of a broader climatological network using the cheaper *SEnviro* sensors. In general, the data generated by low-cost stations do not produce a greater number of inhomogeneities compared to official and professional stations.

## 2 Experimental Design, Materials and Methods

### 2.1 Hardware components and materials

The presented data were collected using *SEnviro* nodes [3]. These nodes are built using open-hardware components and follow the structure defined in [3]. The cited structure defined four different groups Core, Sensing/Acting, Power Supply and Communication. Author in [3] describes each element used to build a *SEnviro* node.

Next list summarizes each component used to build a node:

• **Particle**. It is the main component and acts as the IoT node core. The Particle Electron microcontroller follows an open-source design. It includes a STM32F205RGT6 ARM Cortex M3 chip which operates at a rate of 120 MHz. It can be updated utilising OTA updates. This feature provides a significant increase concerning holding each *SEnviro* node updated and adding new functionalities or functions in the future without the need to move to where the node is deployed physically. The Electron stores variables to carry a regular operation by using 128 KB of RAM and 1 MB of Flash ROM. This microcontroller is built using a chip called MAX17043, which can measure the energy spent by the IoT node. The Particle Electron supports 2G/3G connectivity using a cellular module called U-blox SARA-U270, and it enables an IP connection. A cellular antenna is enclosed for the microcontroller to establish a link to a near cellular tower.
• **Weather shield**. An easy-to-use circuit compatible with the Particle Electron microcontroller is used. This component incorporates two different sensors, the MPL3115A2 (*barometric pressure*) and Si7021 (*relative humidity and temperature*) chips. Moreover, it contains two RJ11 connectors to attach anemometer and rain gauge sensors.
• **Solar panel and lithium-ion battery.** A waterproof solar panel is included to offer an autonomous energy supply. This panel charges the battery and provides a continuous IoT node operation. A lithium-ion battery is included; it holds a 2000 mA capacity and provides an output voltage of 3.7 V.
• **Soil moisture**. This element measures the *moisture of the soil*. It has two pads, and they act as a variable resistor; it gets better conductivity when more water therein in the soil. To obtain the final output (percentage) a re-map function is needed from 0–4096 to 0–100.
• **Weather meters**. An anemometer and rain gauge compose it. They measure the *speed and direction of wind* and *precipitation*. Both sensors are connected using RJ11 connectors.

Following each phenomena collected is described providing units, range and accuracy.

• **Temperature**. Manufacturer: *SparkFun*; Model: *Si7021*; Data Interface: *Analog*; Units: *Centigrade*; Range: *[−10, 85]*; Accuracy: *±0.4 degrees (C)*
• **Humidity**. Manufacturer: *SparkFun*; Model: *Si7021*; Data Interface: *Analog*; Units: *Percentage*; Range: *[0%, 80%]*; Accuracy: *±3 RH*
• **Barometric pressure**. Manufacturer: *SparkFun*; Model: *MPL3115A2*; Data Interface: *I2C*; Units: *Hectopascal*; Range: *[500, 1100]*; Accuracy: *±0.04 hPa*
• **Soil moisture**. Manufacturer: *SparkFun*; Model: *DS18B20*; Data Interface: *Analog*; Units: *Percentage*; Range: *[0%, 85%]*; Accuracy: *±0.5 RH*
• **Wind speed**. Manufacturer: *SparkFun*; Model: *SEN08942*; Data Interface: *Analog (RJ11)*; Units: *km/h*; Range: *N/A;* Accuracy: *N/A*
• **Wind direction**. Manufacturer: *SparkFun*; Model: *SEN08942*; Data Interface: *Analog (RJ11)*; Units: *Direction (degrees)*; Range: *(0) North, (1) NE, (2) East, (3) SE, (4) South, (5) SW, (6) West, (7) NW and (-1) error*; Accuracy: *N/A*
• **Rain meter**. Manufacturer: *SparkFun*; Model: *SEN08942*; Data Interface: *Analog (RJ11)*; Units: *millilitres (mm)*; Range: *(0) North, (1) NE, (2) East, (3) SE, (4) South, (5) SW, (6) West, (7) NW and (-1) error*; Accuracy: *N/A*
• **Battery**. Manufacturer: *N/A*; Model: *N/A*; Data Interface: *N/A*; Units: *Percentage*; Range: *[0%, 100%]*; Accuracy: *N/A*

### 2.2 Deployment

**Fig. 1:**
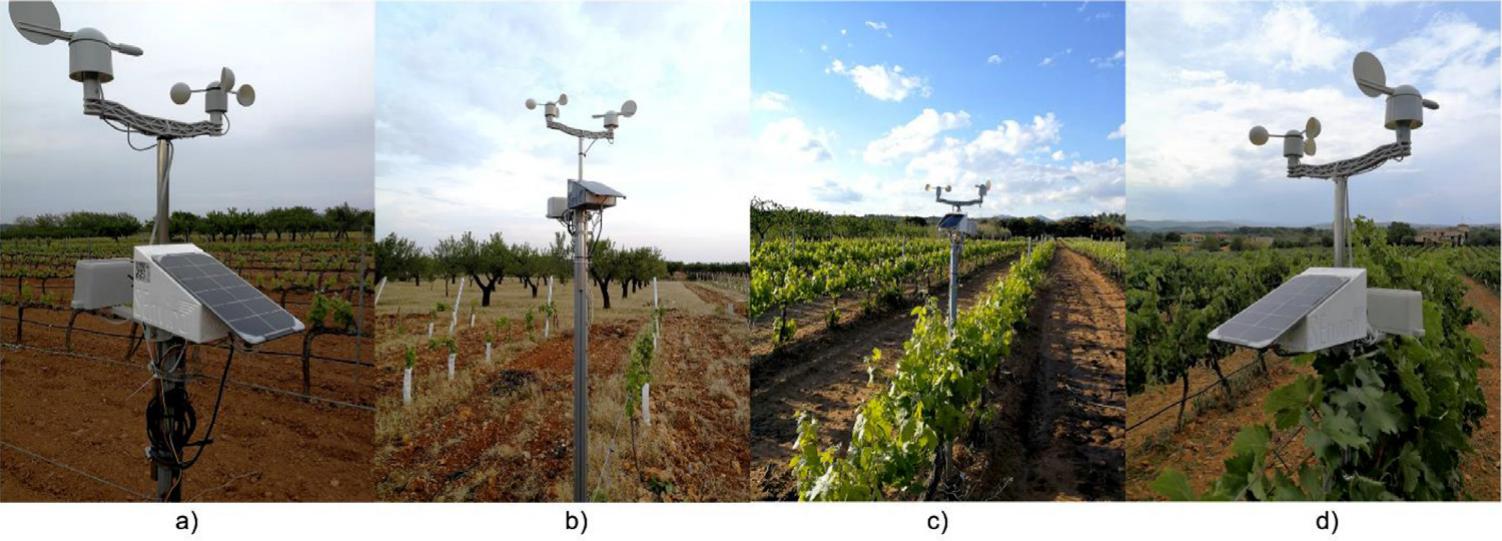
A set of images for each SEnviro node deployment in vineyard smallholdings. In Table 1 (a) Sensor Id. 270043001951343334363036, (b) SEnviro Id.. 4e0031000251353337353037, (c) SEnviro Id.. 380033001951343334363036, (d) SEnviro Id.. 46005a000351353337353037.

**Fig. 2:**
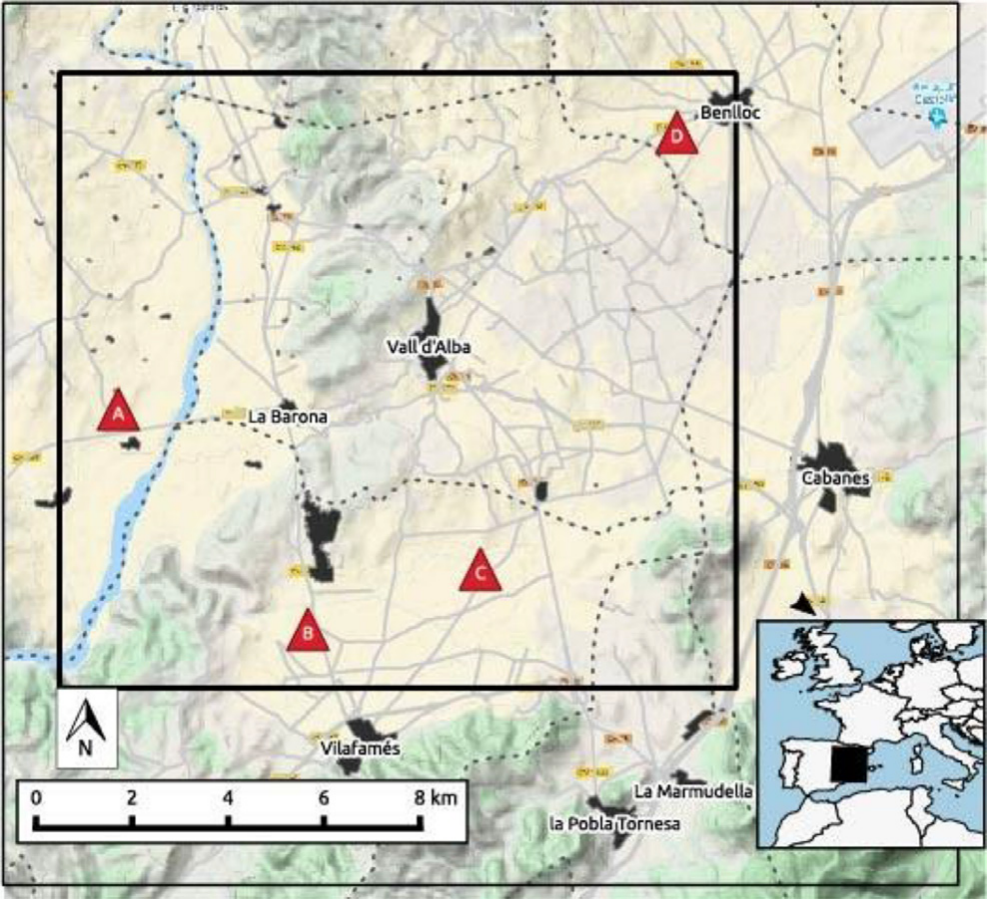
Map showing each of SEnviro nodes locations: (A) SEnviro Id.. 46005a000351353337353037, (B) SEnviro Id. 270043001951343334363036, (C) SEnviro Id.. 380033001951343334363036 and (D) SEnviro Id.. 4e0031000251353337353037.

Seven units of the *SEnviro* node have been deployed; four nodes have been placed in vineyard smallholdings in the province of Castelló (Figs. 1 and 2). All nodes are included in a circular bounding box with a diameter of 4 kilometres. The study area is between 240 to 320 m above sea level. The predominant climate is local steppe. There is little rainfall throughout the year. This climate is considered BSk according to the Köppen-Geiger climate classification. It should be noted that the province of Castelló is an area of great climatic variety due to its mountainous orography [7,8], with high temperatures in summer and very low ones in winter, and an important difference between the coast and the interior. The average temperature in this zone is 14.00 degrees (C) and the approximate precipitation is 434 mm. Next, some details about each vineyard smallholding are provided:

• *SEnviro* Id.: *270043001951343334363036*; Location (lat, lon): *40.133098, −0.061000*; Area (Metres2): *20,000; Grape Variety: Monastrell.*
• *SEnviro* Id.: *380033001951343334363036*; Location (lat, lon): *40.206870, 0.015536*; Area (Metres2): *18,000*; Grape Variety: *Cabernet, Syrah, Merlot and Chardonnay.*
• *SEnviro* Id.: *4e0031000251353337353037*; Location (lat, lon): *40.141384, −0.026397*; Area (Metres2): *15,000*; Grape Variety: *Bonicaire.*
• *SEnviro* Id.:*46005a000351353337353037*; Location (lat, lon): *40.167529, −0.097165*; Area (Metres2): *20,000*; Grape Variety: *Merlot, Tempranillo, Cabernet sauvignon, Syrah and others.*

The other three nodes were deployed in outdoor environments for testing proposes. These testing IoT nodes correspond to identifiers *200034001951343334363036, 46004e000251353337353037* and *4e0031000251353337353037*. The location of these nodes was the Universitat Jaume I (lat: 40.1138985, lon: -0.0519082).

P**article sketches (code)**

The *SEnviro* source code developed to collect and send measurements to a main server using 3G connectivity is available in the following repository [9]. The source code is divided into four different parts. The first part realizes the different declarations and initializations. The second block is the setup where all sensors are initialized, and MQTT connection is established. The third part is the loop stage; it defines all the logic of collecting the data and sending it to the server. It is also in charge of establishing different modes of energy consumption. Finally, the last part includes different ancillary functions used in the previous stage.

## CRediT authorship contribution statement

**Sergio Trilles:** Conceptualization, Methodology, Writing - original draft, Project administration, Funding acquisition, Writing - original draft. **Alberto González-Pérez:** Software, Data curation, Writing - review & editing. **Benito Zaragozí:** Formal analysis, Validation, Writing - original draft. **Joaquín Huerta:** Supervision, Writing - review & editing.

## Declaration of Competing Interest

The authors declare that they have no known competing financial interests or personal relationships which have, or could be perceived to have, influenced the work reported in this article.

## References

[1] Trilles Oliver S, González-Pérez A., Huerta Guijarro J. (2019). Adapting models to warn fungal diseases in vineyards using in-field internet of things (IoT) nodes. Sustainability.

[2] Trilles, S. (2020). Enviromental sensor data collected using low-cost IoT nodes (SEnviro) from vineyard smallholdings (Season 2018) (Version 0.0.1). 10.5281/zenodo.3727310

[3] Trilles, S.; González-Pérez, A.; Huerta, J. An IoT proposal for monitoring vineyards called SEnviro for agriculture. In Proceedings of the 8th International Conference on the Internet of Things (IoT ’18). Association for Computing Machinery, New York, NY, USA, Article 20, 1–4. 10.1145/3277593.3277625

[4] Trilles S., González-Pérez A., Huerta J. (2018). A comprehensive IOT node proposal using open hardware. a smart farming use case to monitor vineyards. Electronics.

[5] Mahmood M.A., Seah W.K., Welch I. (2015). Reliability in wireless sensor networks: a survey and challenges ahead. Comput. Netw..

[6] Trilles, S., Juan, P., Díaz-Avalos, C., Ribeiro, S., & Painho, M. (Submitted). Reliability evaluation of a low‐cost climatic Internet of Things node for applications in agriculture. Sensors, MDPI. 10.3390/s20226597 PMC7698867 33218063

[7] Sala J.Q., Chiva E.M., Vázquez M.V.Q. (2018). La elevación de las temperaturas en el norte de la Comunidad Valenciana: valor y naturaleza (1950-2016). Investigaciones geográficas.

[8] González Herrero, S., & Bech, J. (2017). Extreme point rainfall temporal scaling: a long term (1805-2014) regional and seasonal analysis in Spain: extreme point rainfall temporal scaling in Spain.

[9] Trilles S. (2020). SEnviro-Node Code (Version 0.0.1).

